# 2011–2012 Reviewers

**DOI:** 10.1002/ece3.479

**Published:** 2013-01-10

*Ecology and Evolution* would like to thank all of the reviewers whose support has led to the successful beginning and growth of the Journal. We rely on the expertise and judgment of our reviewers and appreciate the time and effort put into these reviews. We also would like to thank the numerous reviewers at our supporter journals whose reviews were passed along via the manuscript transfer program for their hard work. The manuscript transfer program has a goal of reducing the burden on working researchers to complete reviews by sending the previous reviewer comments of papers transferred to *Ecology and Evolution*, creating a seamless peer-review process. Below is a list of the reviewers submitting one or more reviews directly to *Ecology and Evolution* between May 2011 and December 2012.

Abbott, Richard

Adl, Sina

Alexander, Helen

Alexander, Karen

Alexander, Shelley

Allal, Francois

Allnutt, Tom

Als, Thomas

Anderson, Wyatt

Anholt, Bradley

Arandjelovic, Mimi

Archer, Ruth

Arendt, Jeff

Arnold, Mike

Atwell, Jonathan

Avery, Margaret

Avolio, Meghan

Babbitt, Courtney

Backstrom, Niclas

Bailey, Nathan

Barot, Sebastian

Barton, Daniel

Bashey-Visser, Farrah

Bayer, Till

Bayer-Wilfert, Lena

Beachum, Collin

Beebee, Trevor

Benazzo, Andrea

Ben-Horin, Tal

Benzie, John

Bergman, Eva

Best, Alex

Bidartondo, Martin

Bilgrami, Anwar

Bilton, Mark

Bleakley, Bronwyn

Boeing, Wiebke

Bonduriansky, Russell

Bonneaud, Camille

Bonser, Stephen P.

Branstrator, Donn

Bretman, Amanda

Brooks, Robert

Buck, Alain

Buerkle, Alex

Burbrink, Frank

Burg, Theresa

Burri, Reto

Canal, David

Caporale, Lynn

Carberry, Peter

Carling, Matt

Carrión, José S.

Centeno-Cuadros, Alejandro

Chamaillé-Jammes, Simon

Chambers, Lynda

Chang, Scott

Chang, Liang

Chantawannakul, Panuwan

Chao, Anne

Chevin, Luis-Miguel

Chilton, Neil

Chisholm, Ryan

Chung, MyongGi

Collins, Sinead

Collyer, Michael

Cook, Chelsea

Cooper, Idelle

Corlett, Richard

Cortés, Jorge

Cosson, Jean-François

Cotter, Sheena

Cox, Christian

Crutsinger, Gregory

Cullingham, Catherine

Cunningham, Chris

Curry, R. Allen

Dann, Peter

De Jong, Gerdien

de Knegt, Henrik J.

de Mello-Martins, Felipe

De Roode, Jacobus

de Solla, Shane R.

Dexter, Kyle G.

Diaz-Pulido, Guillermo

DiBattista, Joseph

Dinerstein, Eric

Dinsmore, Stephen

DiTommaso, Toni

Duneau, David

Economo, Evan

Edwards, Alasdair

Einum, Sigurd

el Sabaawi, Rana

Ellis, Christopher

Engelbrecht, Bettina

Etges, William

Everaerts, Claude

Fain, Steven

Farrington, Heather

Fenberg, Phillip

Fenton, Brock

Fisher, Paul

Fleming, Richard A.

Ford, Hugh

Formica, Vince

Fox, Charles

Frago, Enric

Frick, Winifred

Fricke, Anna

Fu, Yong-Bi

Gailing, Oliver

Galian, Jose

Gallagher, Rachael

Galpern, Paul

Gao, Kunshan

Garant, Dany

Garcia-Vazquez, Eva

Garnett, Stephen

Garrido, J.

Gerling, Dan

Germino, Matthew

Gibbs, Adrian

Gomez-Mestre, Ivan

Goodnight, Charles

Gordo, Oscar

Gosden, Tom

Gowaty, Patricia

Grabowski, Michal

Graham, Catherine

Grayson, Kristine

Griffiths, Rob

Groot, Astrid

Gurevitch, Jessica

Gustafson, Richard G.

Haase, Martin

Habel, Jan Christian

Han, Richou

Handel, Colleen

Hansen, Per

Harms, Kyle

Harris, James

Harrison, Xavier

Hatfield, Jerry L.

Haudry, Annabelle

Head, Megan

Hegemann, Arne

Heithaus, Michael

Henmi, Yasuhisa

Heuertz, Myriam

Himes, Christopher

Hirel, Bertrand

Hirsch, Ben

Hoban, Sean

Hoffmann, Frederico

Holwell, Greg

House, Clarissa

Huusko, Ari

Ide, Reiko

Illera, Juan Carlos

James, Bell

Jamieson, Ian

Jaramillo, Carlos

Johannesson, Kerstin

Jones, Adam

Kang, Sinkyu

Keller, Irene

Kimball, Bruce A.

Kirkpatrick, Jamie

Kobayashi, Kazuhiko

Koller, Eva

Kolokotronis, Sergios-Orestis

Koseki, Yusuke

Koskella, Britt

Kotlik, Petr

Kozak, Genevieve

Kraak, Michiel H.S.

Krug, Patrick

Küffer, Cristoph

Kunte, Krushnamegh

Kwapich, Christina

Lang, Michael

Lanta, Vojtěch

Laris, Paul

Lavin, Mathew

Lawler, Richard

Lawson, Callum

Lawson Handley, Lori

Lee, Caitlin

Lee, K. P.

Leger, Elizabeth

Les, Don

Lewis, Sara

Liebers-Helbig, Dorit

Lima, Mauricio

Lima, Fernando

Lindstrom, Jan

Lion, Sebastien

Lock, Judith

Logue, Jürg

Lu, Baorang

Luja, Victor

Lukey, James

M.N, Maruthi

Marchini, Gina

Marshall, Rupert

Marsico, Travis

Masly, John

Mathis, Alicia

McCreadle, John

McElfresh, Steven

McGlothlin, Joel

McMahon, Clive

McPeek, Mark

Meadow, James

Medina, Raul

Men, Xingyuan

Messmer, Vanessa

Millar, Melissa

Mills, Scott

Montrose, Tamara

Mowat, Garth

Mueller, Laurence

Müller, Jörg

Murphy, Nicholas

Musiani, Marco

Nagel, Karl-Otto

Neville, Helen

Nicholson, Kirsten

Noonan, Brice

O'Donnell, Jonathan

Okada, Miki

Oomen, Rebekah

Ornelas, Juan Fransisco

Ortego, Joaquin

Ożgo, Małgorzata

Palacios, M.G.

Papadopoulos, Yousef

Papastamatiou, Yannis

Pauli, Harald

Pavey, Chris

Pearson, Clark

Pennington, Toby

Pilastro, Andrea

Pincheira-Donoso, Daniel

Poisot, Timothée

Poysa, Hannu

Presley, Steven

Price, Tom

Pujolar, JM

Qaderi, Mirwais

Quinn, Tom

Radford, Ian

Randall, Joanna

Raupach, Michael

Ravi, Sujith

Reed, Denné

Ribeiro, Alexandre

Richards, Thomas

Riesch, Ruediger

Riginos, C.

Robinson, Stacey

Rohr, Jason

Rolinson, Njal

Rosato, Ezio

Rossetto, Maurizio

Rotenberry, John

Royer, Dana

Ryder, Thomas

Saenz-Agudelo, Pablo

Santos, Mauro

Sarup, Pernille

Scherrer, Daniel

Schrama, Maarten

Schunter, Celia

Sederoff, Ron

Segner, Helmut

Seigel, Richard A.

Sensenig, Ryan

Sequeira, Andrea

Sergio, Fabrizio

Sexton, Jason

Sezen, Uzay

Sharma, M. D.

Shaw, Jon

Shennan, Carol

Shizuka, Dai

Shustack, Daniel

Sinclair, Brent

Sircom, Julie

Smallwood, Peter

Smiseth, Per

Smith, Craig

Sokolow, Susanne

Sousa, Vitor

Sperling, Felix

St. George, Scott

Stankowich, Ted

Stillman, Richard

St-Laurent, Martin-Hughes

Stott, Iain

Stott, Wendylee

Stronen, Astrid

Stukenbrock, Eva

Sullivan, Jack

Szumik, Claudia

Tack, Ayco

Tammaru, Tommas

Tang, Jinyun

Taylor, Tiffany

Telemeco, Rory

Thalmann, Olaf

Therrell, Matt

Trapnell, Dorset

Trenham, P. C.

Trout Fryxell, Rebecca

Turner, Thomas

Turvey, Samuel

Uribe-Aranzábal, María del Carmen

Ursenbacher, Sylvain

van Baalen, Minus

Van Geert, Anja

Vandermeiren, Karine

Vanderpoorten, Alain

Vaughan, Duncan

Vitasse, Yann

Volckaert, Filip

Wagner, Philipp

Wallis, Graham

Waples, Robin

Watts, Phill

Wedekind, Claus

Wennevik, Vidar

Weston, Mike

Widmer, Ivo

Wiencke, Christian

Willard, Debra

Wilson, Rob

Wilson, Paul

Wilson, Ryan

Wu, Jianqiang

Yeates, Gregor

Yoccoz, Nigel

Zane, Lorenzo

Zhao, Zihua

Zhou, Mei Fu

Ziska, Lewis

